# Thoracic Compression Fracture as a Result of Taser^®^ Discharge

**DOI:** 10.5811/cpcem.2017.7.33508

**Published:** 2017-10-03

**Authors:** Aaron C. Tyagi, Alex Gill, Brent Felton

**Affiliations:** Michigan State University, Sparrow Hospital, Department of Emergency Medicine, Lansing, Michigan

## Abstract

A conducted electrical device (CED), usually Taser®, is commonly used by law enforcement officers to aid in the incapacitation of subjects. While CEDs are considered “safe” for use on subjects, adverse events may rarely occur. We report a case of a 23-year-old male presenting with severe back pain following deployment of a CED with resulting acute compression fractures of the thoracic sixth, seventh, and eighth vertebral bodies. To the best of our knowledge, this represents the third case of traumatic injury from CED discharge to be reported in the literature since 1995.

## INTRODUCTION

The Taser^®^ is a commonly-used, conducted electrical device (CED), used by law enforcement officials nationwide to incapacitate subjects by non-lethal means. These devices employ two electrodes to deliver a high-voltage, low-amperage shock resulting in widespread, involuntary muscle contractions halting further purposeful motor activity of the subject. A CED is intended to serve as a non-lethal alternative that provides an increased measure of safety for both law enforcement officials as well as subjects exposed to the electrical shock. However, its use is not without consequence. With the increased prevalence of CEDs among law enforcement and the general public, it is important for the emergency physician to be familiar with the potential adverse outcomes associated with use of these devices. We present a rare case of multiple thoracic compression fractures resulting from a CED shock that adds to the limited body of evidence regarding complications and injuries following CED deployment.

## CASE REPORT

A 23-year-old male presented to a rural emergency department (ED) for evaluation of mid-back pain following electrocution via a CED. This occurred while the patient, an employee of the Department of Corrections, was volunteering as a model to experience deployment of the device. During the demonstration, Taser^®^ leads were placed on the patient’s right shoulder and ankle and were followed by a five-second electrical discharge from the device. Immediately afterward, the patient complained of bilateral flank muscular contractions and severe pain to the mid-back area that was evident upon presentation. There was no loss of consciousness. The patient had no history of seizures, back trauma or fall either prior to or after the event. Past medical, surgical and social histories were non-contributory.

On examination he was in severe distress. Vital signs revealed a blood pressure of 168/100 mmHg, heart rate of 100 beats per minute (bpm), and were otherwise normal. Back examination revealed midline thoracic and bilateral paravertebral tenderness with limited range of motion secondary to pain. Examination of all four extremities revealed full range of motion without motor or sensory deficits. Examination of other systems was unremarkable.

Computed tomography (CT) of the chest with contrast was performed and revealed acute compression fractures of the superior endplates of the sixth, seventh and eighth thoracic vertebrae without retropulsion of any of the spinal fragments (Image). No further injuries were detected and CTs of the abdomen and pelvis were normal. Subsequently, the patient was transferred to a regional Level I trauma center for further care.

Upon examination at the receiving trauma center, vital signs revealed a blood pressure of 153/92 mmHg with a heart rate of 108 bpm. Laboratory investigations revealed a creatine phosphokinase of 607 units/L and a creatine kinase-MB of 8.9 ng/mL. Urine myoglobin was negative. Following consultation with the trauma service, the patient was admitted for further evaluation. Post-admission, neurosurgical evaluation was obtained and the decision was made for non-operative management using a thoraco-lumbar-sacral-orthosis device, physical therapy and pain control. The patient was eventually discharged to home on post-admission day five after adequate pain control was achieved with recommendation for follow-up on outpatient basis.

## DISCUSSION

Reports on the development of CED devices are dated as early as the 1960s (patented in 1972). The device, attributed to National Aeronautics and Space Administration researcher Jack Cover,^1^ was designed for aiding in the “immobilization and capture” of its intended targets.

Since then, the use of CEDs has become nearly ubiquitous among law enforcement agencies. The largest manufacturer is Taser International, and its devices are reportedly being used in approximately 17,800 of the nation’s 18,250 law enforcement agencies.^1^ CEDs in general can be used in either a “push-stun” or “probe” mode. In the push-stun mode, applying direct pressure with the device against a subject’s body, delivers an electric charge. In the probe-mode, compressed nitrogen is used to propel two barbed probes that are designed to hook onto the subject’s skin or clothing. The probes are attached to the device via thin, insulated copper wiring through which the charge is delivered. The most common CED in use today (TASER^®^ X-26) can produce an electric shock of up to 50,000 volts in an open circuit that is delivered in 100 millisecond pulses at 19 hertz (Hz) over the course of five seconds.^2^

Data suggests that using these devices may reduce the likelihood of injury among both subjects and officers during instances where physical force is required.^3^ In 2008,a prospective analysis by Bozeman et al. estimated that among 1,201 Taser^®^ victims, only 0.25% had significant injury (two intracranial injuries from falls and one case of rhabdomyolysis).^4^ Nonetheless, CEDs are not entirely benign. An increasing number of case reports in the literature describe significant adverse outcomes associated with the use of CEDs including cardiac dysrhythmias, puncture injuries to the cranium and eye, and even pharyngeal perforation.^5^–^8^

Based on our literature review, we identified only two cases of vertebral compression fractures resulting from CED deployment that have been reported over the last 10 years.^(9,10)^ Similar to our case, the injuries encountered in both of the these cases resulted from CED deployment without involvement of a secondary injury such as fall or seizure. As such, to the best of our knowledge, this report represents the third case of vertebral compression fractures resulting from CED deployment to date. An interesting observation was that all three cases involved law enforcement officers acting as models during demonstration of the device. Further, the resultant vertebral fractures in all three cases were stable and therefore managed non-operatively. Our report however represents the only case to date to suffer a vertebral body fracture following CED deployment without any identifiable risk for fracture. Radiographic imaging of the two previous case reports demonstrated potential risk factors for fracture or previous injury including diffuse osteopenia in one case, and a history of wedge deformity of the second lumbar vertebrae in the other.

**CPC-EM Capsule**

What do we already know about this clinical entity?

Cases of traumatic injury from a conducted electrical device (CED), most commonly Taser®, have rarely been reported. In patients with known comorbidities such as osteoporosis or smoking history, CED-discharge injury may be more likely than in an otherwise healthy population.

What makes this presentation of disease reportable?

This is the first reported case of spinal fracture from CED discharge in an otherwise healthy patient.

What is the major learning point?

A CED is not a benign entity. Injuries can occur in all populations.

How might this improve emergency medicine practice?

Maintaining a high index of suspicion for injury secondary to CED discharge is paramount to the EM provider, as injury can occur even in the healthy population without any other risk factors or comorbidities.

Of question is the specific mechanism and dynamics resulting in those vertebral fractures. We hypothesize that this may have been due to diffuse and powerful contraction of paraspinous muscles induced by electrical current resulting in compression fractures. The fact that similar vertebral compression fractures have been reported in the literature as a result of brief, accidental electrocutions,^11^,^12^ as well as generalized, tonic-clonic seizure activity,^13^,^14^ supports our hypothesis. In the latter cases, vertebral compression fractures are thought to occur via a mechanism of sudden, severe, paraspinous muscle contractions. Similarly, the electrical impulses emitted by CEDs are designed to stimulate alpha-motor neurons, triggering “powerful, incapacitating levels of skeletal muscle force production.”^15^

## CONCLUSION

Emergency physicians and first responders should be aware of the potential injuries for individuals who have been subjected to deployment of a Taser^®^. While the few documented cases of vertebral injuries after the use of a conducted electrical device have resulted in stable fractures, providers should still take the appropriate precautions when assessing and transporting CED victims complaining of back pain or parasthesias. Emergency physicians should consider radiographic studies to assess for vertebral fractures in patients complaining of back pain or tenderness on exam following CED deployment. Vertebral body fractures represent a rare but clinically significant adverse event that can occur with the use of CEDs.

## Figures and Tables

**Image:**
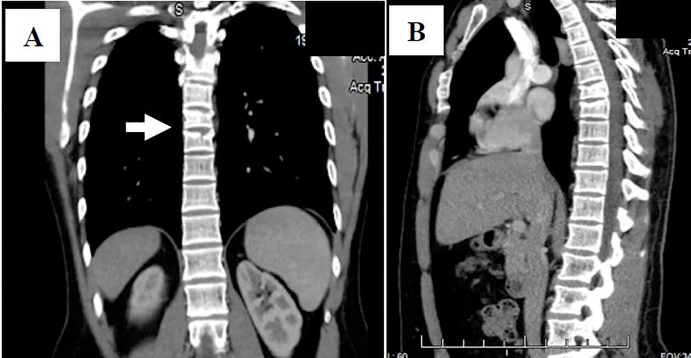
**(A)** Computed tomography of the chest (coronal view) showing fracture deformities of the thoracic 6th – 8th vertebral bodies (white arrow); **(B)** Sagittal view.

## References

[1] Soleimanirahbar A, Lee BK (2011). The TASER safety controversy. Expert Rev Med Devices.

[2] (2011). Justice NIo. Study of Deaths Following Electro Muscular Disruption.

[3] Smith MR, Kaminski RJ, Alper GP (July). A Multi-Method Evaluation of Police Use of Force Outcomes: Final Report to the National Institute of Justice.

[4] Bozeman WP, Hauda WE, Heck JJ (2009). Safety and injury profile of conducted electrical weapons used by law enforcement officers against criminal suspects. Ann Emerg Med.

[5] Kim PJ, Franklin WH (2005). Ventricular fibrillation after stun-gun discharge. N Engl J Med.

[6] Le Blanc-Louvry I, Gricourt C, Toure E (2012). A brain penetration after Taser injury: controversies regarding Taser gun safety. Forensic Sci Int.

[7] Ng W, Chehade M (2005). Taser penetrating ocular injury. Am J Ophthalmol.

[8] Al-Jarabah M, Coulston J, Hewin D (2008). Pharyngeal perforation secondary to electrical shock from a Taser gun. Emerg Med J.

[9] Winslow JE, Bozeman WP, Fortner MC (2007). Thoracic compression fractures as a result of shock from a conducted energy weapon: a case report. Ann Emerg Med.

[10] Sloane CM, Chan TC, Vilke GM (2008). Thoracic spine compression fracture after TASER activation. J Emerg Med.

[11] Sinha A, Dholakia M (2009). Thoracic compression fracture caused by electrically induced injury. PM R.

[12] van den Brink WA, van Leeuwen O (1995). Lumbar burst fracture due to low voltage shock. A case report. Acta Orthop Scand.

[13] Takahashi T, Tominaga T, Shamoto H (2002). Seizure-induced thoracic spine compression fracture: case report. Surg Neurol.

[14] Roohi F, Fox A (2006). Burst fracture of the first lumbar vertebra and conus-cauda syndrome complicating a single convulsive seizure: a challenge of diagnosis in the Emergency Department. J Emerg Med.

